# The National Medical Union: Meeting of the Provisional Council

**Published:** 1913-12-20

**Authors:** R. Carswell, E. A. Dowell


### The National Medical Union.

### A NON-PANEL REGISTER.

A meeting of the Provisional Federating Council was held at the Waldorf Hotel on December 11 at 4.30 p.m. Mr. Damer Harrisson, of Liverpool, was in the chair. Much business was transacted, and genuine enthusiasm for the cause was displayed. The following important matters were agreed to:—

### A Preliminary Guarantee Fund.

The first financial year will extend from December 1, 1913, to December 1, 1914. A guarantee fund (voluntary) with a minimum of £200 was formed, and before the end of the meeting this fund amounted to £165. All willing to subscribe to it are invited to send in their names and subscriptions at once to one of the hon. treasurers, Dr. Brassey Brierley (Manchester) or Dr. H. Oppenheimer (Hampstead). It was further determined that the liability of the guarantors of this preliminary guarantee fund should expire on the 1st of April, 1914.

### An Executive Committee.

It was resolved to appoint an Executive Committee to consist of eleven members in addition to ex-officio members of Council, this executive to be formed from the Council.

The following members were elected on this Committee:—Dr. Sharman of Hampstead, Dr. Greene of Wandsworth, Dr. Pinder of Manchester, Dr. Westmacott of Manchester, Dr. Lowe of Crewe, Dr. Hemmans of Lewisham, Dr. Spurgin of Marylebone, Dr. Damer Harrisson of Liverpool, Dr. O'Sullivan of Liverpool, Dr. Thyne of Edinburgh, and Dr. Johnston of Brighton.

### Finance and Press Committees.

A Finance Committee was appointed consisting of the following:—Dr. Ford Anderson (Hampstead), Dr. Porter (Edinburgh), and Dr. Westmacott (Manchester).

A Press Committee was formed consisting of:—Dr. Greene (Wandsworth), Dr. Skardon Prowse (Manchester), and Dr. James Smith (Edinburgh).

### Further Resolutions.

7. "That in the event of any member of the Executive or any other Committee being unable to attend a meeting a deputy who is already a member of Council may be appointed to attend in his place."

8. "That an advertisement be inserted in the press inviting all non-panel medical men to communicate with the hon. secretaries of the Union."

9. "That it be the policy of the Council to promote non-panel associations according to the distribution of the Insurance areas."

10. "That it be referred to the Executive Committee to draw up a memorandum of policy to be presented to the next meeting of Council."

The question of the formation of a complete register of non-panel practitioners was discussed, together with a number of other matters appertaining to the initial steps of organisation.

### Executive and Sub-Committees.

Meetings of the Executive and sub-Committees are to take place on the 20th inst., when a number of matters appertaining to correspondence, clerical assistance, offices, matters of policy, and press arrangements will come up for consideration.

R. Carswell.
E. A. Dowell.

